# Precision Approaches in Diabetes Prevention: Towards a Clustering Approach With More Phenotypical Granularity

**DOI:** 10.1210/clinem/dgaf341

**Published:** 2025-06-13

**Authors:** Ellen E Blaak

**Affiliations:** Department of Human Biology, Maastricht University, NUTRIM Institute for Nutrition and Translational Research in Metabolism, Maastricht University, P.O. Box 616, Maastricht 6200 MD, The Netherlands

**Keywords:** precision nutrition, precision medicine, prediabetes, clustering approach, phenotypic granularity

Prediabetes and type 2 diabetes (T2D) are major global health concerns. Current treatment or prevention strategies do not take individual metabolic variability into account. An increased understanding of the different etiologies towards T2D, the different prediabetes phenotypes, may improve risk stratification and may be the basis for more precision-based prevention strategies. Recent studies have identified subphenotypes of T2D and prediabetes (1), which may differ in risk for disease progression, severity, and possibly in response to interventions. So far, most studies have focused on individuals with T2D and not on prediabetes while the prediabetic state may provide the window of opportunity to prevent T2D. Additionally, few studies have explored granular markers of body composition (eg, adipose tissue distribution and ectopic fat depots).

The article by Stroebel et al (2) executes a post hoc analysis on prediabetes phenotypes in the Diabetes Prevention trial, which compared lifestyle intervention and metformin to placebo (conventional treatment) for T2D prevention. In the original trial, the intervention was successful with metformin, and lifestyle intervention resulted in a reduced T2D incidence of 31% and 58%, respectively (3). In the current study, they applied clustering methods to identify prediabetes phenotypes using standard clinical phenotyping (Clinical model) as well as a model where more granular body composition measures were added (ClinicalPLUS + model) and evaluated their relationships with treatment arms and T2D progression (2).

The study identified 5 distinct clusters in both the Clinical and the ClinicalPLUS + models, with each model having different levels of individual risk factors. In the Clinical model, clusters were labeled as older, dyslipidemia, insulin resistant, younger protected, and higher adiposity. In the ClinicalPLUS + model, clusters were labeled as hepatic steatosis, dyslipidemia–insulin resistance, subcutaneous adipose, protected, and older dysglycemia. Both models showed significant differences in time to T2D progression across clusters. The insulin-resistant cluster (Clinical model) and older dysglycemia cluster (ClinicalPLUS + model) had the highest risk for T2D progression, while the younger protected (Clinical model) and protected (ClinicalPLUS + model) clusters had the lowest risk. The higher granularity in the body composition measures included in the Stroebel analysis showed more distinct obesity phenotypes. Of these phenotypes, the subcutaneous adipose tissue phenotype had a longer time to diabetes relative to the dyslipidemia–insulin resistance cluster. Therefore, the addition of more refined estimates of different adipose tissue depots confirmed what prior studies demonstrated: that type of adipose tissue, and not just body mass index (BMI), differentiates risk for T2D. Additionally, the subcutaneous adipose tissue and the protected phenotype cluster had a higher percentage of women (around 85%). This may reflect the different cardiometabolic risk profile in women compared with men, with the former group having a higher proportion of subcutaneous adipose tissue (in particular gynoid adipose tissue) that may be protective in view of its larger reserve to store fat and protect other organs from lipid overflow. The differential T2D risk based on baseline phenotype highlights the potential of better capturing prediabetes risk, which may give leads for a targeted treatment. Indeed, metformin showed to some extent varying efficacy across clusters, with greater impact in clusters characterized by cardiometabolic risk factors other than insulin resistance and dysglycemia.

Nevertheless, the differential intervention effects for baseline phenotypes were not seen for the lifestyle intervention arm; lifestyle was equally effective across all phenotypes. This may be somewhat surprising since there are strong indications that the lifestyle intervention may also depend on initial metabolic phenotype. As an example of a proof of concept study in the field, recently distinct metabolic phenotypes were identified among individuals with overweight, characterized by tissue-specific insulin resistance in either liver (LIR) or muscle (MIR). These metabotypes showed unique metabolomic, lipidomic, and transcriptomic profiles (4). Dietary intervention (PERSON study, n = 242, 58% females) focused on modulation of macronutrient composition tailored to isolated LIR or MIR led to improved insulin sensitivity and cardiometabolic health, independent of weight loss, demonstrating the potential of precision nutrition (5). MIR and LIR are already present in the overweight state and may translate into phenotypes of isolated impaired fasting glucose and impaired glucose tolerance, characterized by LIR and MIR, respectively, among other factors. Thus, lifestyle intervention based on initial phenotypes may increase intervention efficacy. The reason why this is not seen in the Stroebel analyses may possibly relate to the included clustering factors and to the granularity of phenotypic factors included as well as the granularity of dietary intervention. Indeed, inclusion of detailed body composition measures led to more distinct obesity phenotypes with a differential risk for T2D (3), illustrating that it is important to go beyond body fat and focus on body fat distribution and type of adipose tissue. This raises the question whether for defining clusters that represent different etiologies towards cardiometabolic disease, the basis for a differential response to lifestyle intervention, we may have to aim for a more detailed phenotype and go further beyond BMI and age. In this respect it may be of importance to also include, for instance, reliable measures of liver fat and muscle fat accumulation, as well as postprandial measures of glucose and insulin metabolism, possibly representing tissue-specific effects. Interestingly, it may be relevant that distinct etiologies towards cardiometabolic health outcomes have been shown for discordant visceral and liver fat phenotypes (6), discordant liver and muscle fat/mass phenotypes (7), as well as phenotypes discordant for indices of adipose tissue and whole-body insulin resistance (8). It is of great importance to further study how stable these phenotypes are and how they evolve over time.

Overall, the study of Stroebel et al (2) shows that data-driven clustering of prediabetic subgroups allows identification of phenotypes at greater risk for T2D and response to interventions (metformin). Additionally, more granular phenotypic variables included in the clustering approach with respect to subcutaneous and visceral fat accumulation may be of importance to identify different etiologies towards cardiometabolic disease. This may be a crucial step towards phenotype-based intervention strategies to prevent T2D. For the latter, more detailed phenotypic variables may have to be included where we go beyond classical variable like BMI and age and possibly include variables related to tissue-specific and postprandial metabolism.

## Disclosures

E.E.B. has no conflict of interest to declare.

## Abbreviations

BMI: body mass index
LIR: liver insulin resistance
MIR: muscle insulin resistance
T2D: type 2 diabetes
